# *catena*-Poly[[[bis­(2,2′-bipyridine)manganese(II)]-μ_4_-3,3′-sulfanediyldipropionato] bis­(perchlorate)]

**DOI:** 10.1107/S1600536810008123

**Published:** 2010-03-06

**Authors:** Li Yong

**Affiliations:** a Suzhou Vocational University, Suzhou 215104, People’s Republic of China

## Abstract

The title compound, [Mn_2_(C_6_H_8_O_4_S)(C_10_H_8_N_2_)_4_](ClO_4_)_2_, which was crystallized from an aqueous solution, features two Mn^II^ atoms in the asymmetric unit, each being coordinated by four N-atom donors from 2,2′-bipyridine ligands and two O atoms of two different 3,3′-sulfanediyldipropionate (*L*) ligands, with the O atoms in *cis* positions. The two carboxyl­ate groups of each *L* ligand, which adopt a *syn-anti* coordination mode, combine with four Mn^II^ atoms, yielding one-dimensional chains extending along [010].

## Related literature

For the structures and potential applications of metal-organic coordination polymers, see: Gardner *et al.* (1995 ▶); Seo *et al.* (2000 ▶). Many ligands, including rigid carboxyl­ate arms, have been used in the design of metal-organic materials with desired topologies, see: Cao *et al.* (2002 ▶); Xu *et al.* (2005 ▶). Relatively fewer complexes have been reported derived from flexible carboxyl­ate arms, see: Cao *et al.* (2004 ▶); Yong *et al.* (2004 ▶). For the corresponding zinc(II) and cadmium(II) complexes, see: Yang *et al.* (2008 ▶). 
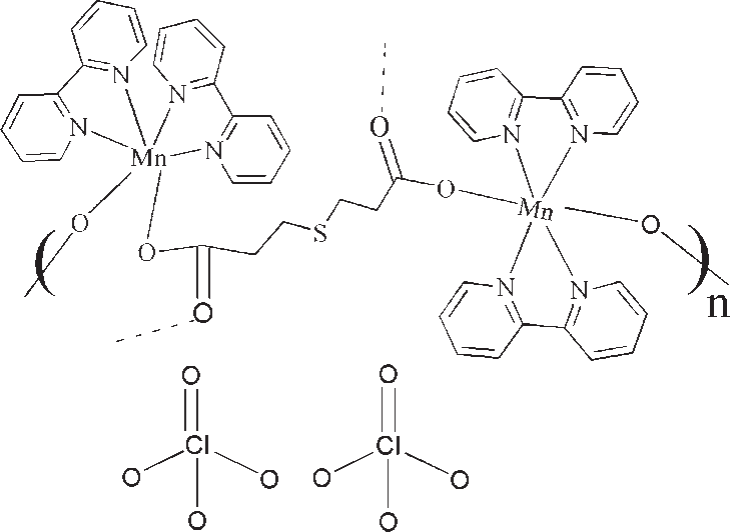


## Experimental

### 

#### Crystal data

[Mn_2_(C_6_H_8_O_4_S)(C_10_H_8_N_2_)_4_](ClO_4_)_2_
*M* *_r_* = 1109.70
Triclinic, 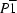

*a* = 12.031 (2) Å
*b* = 13.550 (2) Å
*c* = 15.653 (3) Å
α = 102.611 (2)°
β = 93.213 (3)°
γ = 93.556 (3)°
*V* = 2479.2 (7) Å^3^
*Z* = 2
Mo *K*α radiation
μ = 0.73 mm^−1^
*T* = 294 K
0.22 × 0.16 × 0.10 mm

#### Data collection

Bruker SMART CCD area-detector diffractometer
Absorption correction: multi-scan (*SADABS*; Sheldrick, 1996 ▶) *T*_min_ = 0.841, *T*_max_ = 0.988
12678 measured reflections
8690 independent reflections
5935 reflections with *I* > 2σ(*I*)
*R*_int_ = 0.021

#### Refinement

*R*[*F* ^2^ > 2σ(*F* ^2^)] = 0.043
*wR*(*F* ^2^) = 0.116
*S* = 1.02
8690 reflections
640 parameters
H-atom parameters constrained
Δρ_max_ = 0.53 e Å^−3^
Δρ_min_ = −0.37 e Å^−3^

### 

Data collection: *SMART* (Bruker, 2007 ▶); cell refinement: *SAINT* (Bruker, 2007 ▶); data reduction: *SAINT*; program(s) used to solve structure: *SHELXS97* (Sheldrick, 2008 ▶); program(s) used to refine structure: *SHELXL97* (Sheldrick, 2008 ▶); molecular graphics: *SHELXL97*; software used to prepare material for publication: *SHELXTL* (Sheldrick, 2008 ▶) and local programs.

## Supplementary Material

Crystal structure: contains datablocks I, global. DOI: 10.1107/S1600536810008123/fi2094sup1.cif

Structure factors: contains datablocks I. DOI: 10.1107/S1600536810008123/fi2094Isup2.hkl

Additional supplementary materials:  crystallographic information; 3D view; checkCIF report

## Figures and Tables

**Table 1:** Selected bond lengths (Å)

Mn1—O2^i^	2.121 (2)
Mn1—O1	2.124 (2)
Mn1—N1	2.234 (3)
Mn1—N4	2.236 (3)
Mn1—N3	2.254 (3)
Mn1—N2	2.293 (3)
Mn2—O4^ii^	2.110 (2)
Mn2—O3	2.112 (2)
Mn2—N8	2.255 (3)
Mn2—N6	2.263 (3)
Mn2—N5	2.298 (3)
Mn2—N7	2.301 (3)

Symmetry codes: (i) 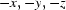; (ii) 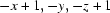.

**Fig. 1.**
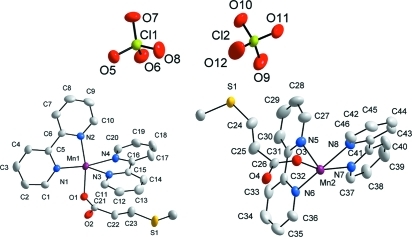
The asymmetric unit of the title compound, showing the atom-labelling scheme. For the sake of clarity, hydrogen atoms have been omitted.

**Fig. 2.**
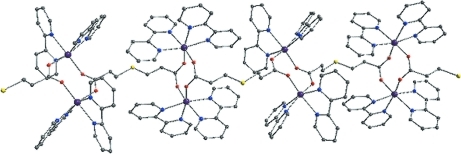
The one-dimensional infinate chain in the compound: for the sake of clarity, hydrogen atoms and ClO4 anions have been omitted.

## supplementary crystallographic information

### Comment

In recent years, a great deal of effort has been devoted to metal-organic coordination polymers owning to their structures and potential applications. (Gardner *et al.*, 1995; Seo *et al.*, 2000;) Many ligands including rigid carboxylate arms have been used in the design of metal-organic materials with desired topologies (Cao *et al.*, 2002; Xu *et al.*, 2005). Compared to rigid carboxylate arms, relative fewer complexes have been reported derived from flexible carboxylate arms (Cao *et al.*, 2004; Yong *et al.*, 2004). We know that the formation of crystal structure is sensitive to the flexibility of carboxylate arms, and such flexibility may increase the probability of making tube-like or cage-like structure. Herein, in the course of ongoing studies on the exploration on carboxylate ligand with flexible carboxylate arms, we choose a multi-carboxylate ligand, namely 3,3'-thiodipropionic acid, which can act not only as chelating ligand, but also as bridging ligand, to assemble new coordination polymers. In this contribution, in the hope of obtaining a supramolecular complex, we used 3,3'-thiodipropionic acid reacting with manganese salts as well as 2,2'-bipyridine ligands and synthesized a new one-dimensional complex. Here, we report the preperation and X-ray characterization of the complex, namely (Mn_2_*L*(bpy)_4_).(ClO_4_)_2_.

The molecular structure of the complex is depicted in Figure 1. In the complex, the local coordination of the manganese atom are defined by four nitrogen donors from bpy ligands with Mn···N distance from 2.236 (2) to 2.293 (2)Å and by two oxygen atoms of different *L* ligands with Mn···O distance from 2.124 (2) to 2.121 (6) Å, yielding a distorted octahedral geometry. Two carboxylic groups of each *L* ligand, which adopt *syn*-anti coordination mode, combine with four manganese(II) atoms to form a one-dimensional chain (shown in Figure 2). The distance of two manganese(II) atom bridged by *L* ligand is 4.7693 Å. Figure 3 shows the molecular packing diagram of the complex viewed in the *ac* plane. In the complex, two carboxylic groups and the sulfur atom of each *L* ligand adopt *syn*-anti conformation to debase the steric hindrance.

### Experimental

A solution of Mn(ClO_4_)_2.(H_2_O)_6 (0.365 g, 1 mmol) was slowly added to a mixture of *L* ligand (0.180 g, 1 mmol) and NaOH (0.040 g, 1 mmol) in water (10 ml) with stirring under heating. Then a solution of bpy (0.198 g, 1 mmol) in ethanol (5 ml) was dropped into the mixture. The resulting solution was continued with stirring for another 2 h under heating and filtered when cooled to room temperature. The yellow crystals suitable for X-ray crystallographic analysis were obtained after a week. The product was washed with cold water and air-dried. Analysis found: C 49.65, H 3.49, N 9.91%; C_46_H_40_Cl_2_Mn_2_N_8_O_12_S requires: C 49.74, H 3.60, N 10.09%.

### Refinement

Crystals of the title complex are triclinic, space group P-1. All non-hydrogen atoms were refined anisotropically. Hydrogen atoms were visible in difference maps and were subsequently treated as riding atoms with distances restraints of C—H = 0.97 (CH_2_) or 0.93Å (CH).

### Crystal data

**Table:**

[Mn_2_(C_6_H_8_O_4_S)(C_10_H_8_N_2_)_4_](ClO_4_)_2_	*Z* = 2
*M**_r_* = 1109.70	*F*(000) = 1136
Triclinic, *P*1	*D*_x_ = 1.487 Mg m^−^^3^
Hall symbol: -P 1	Mo *K*α radiation, λ = 0.71073 Å
*a* = 12.031 (2) Å	Cell parameters from 4396 reflections
*b* = 13.550 (2) Å	θ = 2.5–25.9°
*c* = 15.653 (3) Å	µ = 0.73 mm^−^^1^
α = 102.611 (2)°	*T* = 294 K
β = 93.213 (3)°	Block, yellow
γ = 93.556 (3)°	0.22 × 0.16 × 0.10 mm
*V* = 2479.2 (7) Å^3^	

### Data collection

**Table:**

Bruker SMART CCD area-detector diffractometer	8690 independent reflections
Radiation source: fine-focus sealed tube	5935 reflections with *I* > 2σ(*I*)
graphite	*R*_int_ = 0.021
phi and ω scans	θ_max_ = 25.0°, θ_min_ = 1.3°
Absorption correction: multi-scan (*SADABS*; Sheldrick, 1996)	*h* = −8→14
*T*_min_ = 0.841, *T*_max_ = 0.988	*k* = −16→14
12678 measured reflections	*l* = −17→18

### Refinement

**Table:**

Refinement on *F*^2^	0 restraints
Least-squares matrix: full	H-atom parameters constrained
*R*[*F*^2^ > 2σ(*F*^2^)] = 0.043	*w* = 1/[σ^2^(*F*_o_^2^) + (0.0476*P*)^2^ + 1.3954*P*] where *P* = (*F*_o_^2^ + 2*F*_c_^2^)/3
*wR*(*F*^2^) = 0.116	(Δ/σ)_max_ = 0.001
*S* = 1.02	Δρ_max_ = 0.53 e Å^−^^3^
8690 reflections	Δρ_min_ = −0.37 e Å^−^^3^
640 parameters	

### Special details

**Table:**

Geometry. All esds (except the esd in the dihedral angle between two l.s. planes) are estimated using the full covariance matrix. The cell esds are taken into account individually in the estimation of esds in distances, angles and torsion angles; correlations between esds in cell parameters are only used when they are defined by crystal symmetry. An approximate (isotropic) treatment of cell esds is used for estimating esds involving l.s. planes.

### Fractional atomic coordinates and isotropic or equivalent isotropic displacement parameters (Å^2^)

**Table:**

	*x*	*y*	*z*	*U*_iso_*/*U*_eq_	
Mn1	0.00691 (4)	0.18036 (4)	0.04401 (3)	0.04195 (14)	
Mn2	0.44247 (4)	0.13551 (4)	0.60030 (3)	0.04089 (14)	
S1	0.01371 (9)	0.00873 (11)	0.36605 (7)	0.0797 (4)	
O1	−0.09407 (19)	0.05450 (17)	0.06451 (15)	0.0516 (6)	
O2	−0.0797 (2)	−0.10541 (18)	0.07046 (15)	0.0576 (6)	
O3	0.3690 (2)	0.00206 (17)	0.51458 (15)	0.0538 (6)	
O4	0.3952 (2)	−0.08857 (19)	0.38353 (16)	0.0603 (7)	
N1	−0.1243 (2)	0.2224 (2)	−0.04545 (17)	0.0447 (7)	
N2	0.0733 (2)	0.3260 (2)	0.00598 (18)	0.0482 (7)	
N3	−0.0269 (2)	0.2710 (2)	0.17757 (17)	0.0470 (7)	
N4	0.1538 (2)	0.1728 (2)	0.13582 (18)	0.0494 (7)	
N5	0.2905 (2)	0.2255 (2)	0.58200 (19)	0.0537 (8)	
N6	0.4855 (2)	0.2419 (2)	0.51181 (17)	0.0474 (7)	
N7	0.4777 (2)	0.2644 (2)	0.72353 (17)	0.0462 (7)	
N8	0.3891 (2)	0.0761 (2)	0.71663 (18)	0.0495 (7)	
C1	−0.2205 (3)	0.1664 (3)	−0.0711 (2)	0.0548 (9)	
C2	−0.2844 (3)	0.1696 (3)	−0.1456 (3)	0.0624 (10)	
C3	−0.2485 (4)	0.2349 (3)	−0.1955 (3)	0.0672 (11)	
C4	−0.1508 (3)	0.2953 (3)	−0.1695 (2)	0.0579 (10)	
C5	−0.0893 (3)	0.2875 (2)	−0.0941 (2)	0.0437 (8)	
C6	0.0169 (3)	0.3484 (2)	−0.0628 (2)	0.0455 (8)	
C7	0.0578 (4)	0.4254 (3)	−0.1006 (3)	0.0668 (11)	
C8	0.1570 (4)	0.4800 (3)	−0.0660 (3)	0.0820 (14)	
C9	0.2121 (4)	0.4584 (3)	0.0051 (3)	0.0768 (13)	
C10	0.1686 (3)	0.3801 (3)	0.0388 (3)	0.0626 (10)	
C11	−0.1235 (3)	0.3113 (3)	0.1952 (2)	0.0567 (9)	
C12	−0.1491 (4)	0.3540 (3)	0.2788 (3)	0.0703 (11)	
C13	−0.0740 (4)	0.3528 (3)	0.3471 (3)	0.0794 (13)	
C14	0.0255 (4)	0.3107 (3)	0.3300 (2)	0.0700 (12)	
C15	0.0481 (3)	0.2711 (2)	0.2442 (2)	0.0485 (8)	
C16	0.1523 (3)	0.2232 (3)	0.2196 (2)	0.0502 (9)	
C17	0.2448 (4)	0.2287 (4)	0.2772 (3)	0.0803 (13)	
C18	0.3369 (4)	0.1801 (4)	0.2501 (3)	0.0860 (14)	
C19	0.3375 (3)	0.1271 (3)	0.1658 (3)	0.0691 (11)	
C20	0.2449 (3)	0.1260 (3)	0.1106 (3)	0.0602 (10)	
C21	−0.0823 (3)	−0.0139 (3)	0.1054 (2)	0.0412 (8)	
C22	−0.0775 (3)	0.0126 (3)	0.2050 (2)	0.0553 (9)	
C23	0.0177 (3)	−0.0257 (3)	0.2481 (2)	0.0691 (11)	
C24	0.1392 (4)	−0.0405 (4)	0.4038 (3)	0.0852 (14)	
C25	0.2439 (4)	0.0136 (3)	0.3925 (3)	0.0745 (12)	
C26	0.3433 (3)	−0.0280 (3)	0.4341 (2)	0.0498 (9)	
C27	0.1953 (3)	0.2153 (3)	0.6200 (3)	0.0751 (12)	
C28	0.1122 (4)	0.2822 (4)	0.6202 (4)	0.0940 (16)	
C29	0.1298 (5)	0.3605 (4)	0.5792 (4)	0.0967 (17)	
C30	0.2265 (4)	0.3717 (3)	0.5399 (3)	0.0782 (13)	
C31	0.3061 (3)	0.3027 (3)	0.5413 (2)	0.0525 (9)	
C32	0.4114 (3)	0.3080 (2)	0.4981 (2)	0.0490 (9)	
C33	0.4348 (4)	0.3754 (3)	0.4449 (3)	0.0692 (12)	
C34	0.5352 (5)	0.3734 (4)	0.4064 (3)	0.0834 (14)	
C35	0.6093 (4)	0.3076 (4)	0.4220 (3)	0.0805 (13)	
C36	0.5824 (4)	0.2432 (3)	0.4752 (2)	0.0620 (10)	
C37	0.5167 (3)	0.3587 (3)	0.7230 (2)	0.0566 (10)	
C38	0.5160 (4)	0.4381 (3)	0.7933 (3)	0.0721 (12)	
C39	0.4740 (5)	0.4204 (3)	0.8676 (3)	0.0883 (15)	
C40	0.4348 (4)	0.3239 (3)	0.8707 (2)	0.0753 (13)	
C41	0.4382 (3)	0.2461 (3)	0.7976 (2)	0.0489 (8)	
C42	0.4041 (3)	0.1390 (3)	0.7961 (2)	0.0469 (8)	
C43	0.3942 (3)	0.1036 (3)	0.8722 (2)	0.0603 (10)	
C44	0.3718 (3)	0.0017 (3)	0.8659 (3)	0.0687 (11)	
C45	0.3576 (3)	−0.0629 (3)	0.7856 (3)	0.0675 (11)	
C46	0.3654 (3)	−0.0226 (3)	0.7124 (3)	0.0628 (10)	
Cl1	0.55665 (8)	0.29282 (7)	0.09891 (6)	0.0576 (2)	
Cl2	0.81938 (9)	0.43090 (8)	0.63234 (7)	0.0635 (3)	
O5	0.4477 (2)	0.3230 (2)	0.08365 (18)	0.0736 (8)	
O6	0.5608 (3)	0.1882 (2)	0.0577 (2)	0.0859 (9)	
O7	0.6353 (3)	0.3516 (2)	0.0625 (2)	0.0992 (11)	
O8	0.5816 (3)	0.3050 (3)	0.19051 (19)	0.0975 (10)	
O9	0.7915 (4)	0.3273 (3)	0.6220 (3)	0.1298 (16)	
O10	0.8648 (3)	0.4539 (3)	0.5569 (2)	0.0994 (11)	
O11	0.9012 (3)	0.4606 (3)	0.7031 (2)	0.1141 (13)	
O12	0.7260 (4)	0.4848 (4)	0.6470 (3)	0.1559 (19)	
H1	−0.2455	0.1229	−0.0366	0.066*	
H2	−0.3504	0.1286	−0.1617	0.075*	
H3	−0.2897	0.2384	−0.2467	0.081*	
H4	−0.1264	0.3410	−0.2022	0.069*	
H7	0.0189	0.4401	−0.1487	0.080*	
H8	0.1859	0.5313	−0.0913	0.098*	
H9	0.2777	0.4959	0.0303	0.092*	
H10	0.2072	0.3641	0.0866	0.075*	
H11	−0.1757	0.3105	0.1489	0.068*	
H12	−0.2165	0.3832	0.2887	0.084*	
H13	−0.0899	0.3802	0.4046	0.095*	
H14	0.0774	0.3088	0.3758	0.084*	
H17	0.2443	0.2656	0.3347	0.096*	
H18	0.3989	0.1832	0.2891	0.103*	
H19	0.3989	0.0926	0.1462	0.083*	
H20	0.2454	0.0908	0.0525	0.072*	
H22A	−0.0738	0.0858	0.2249	0.066*	
H22B	−0.1462	−0.0146	0.2238	0.066*	
H23A	0.0870	0.0025	0.2312	0.083*	
H23B	0.0150	−0.0989	0.2289	0.083*	
H24A	0.1354	−0.0397	0.4657	0.102*	
H24B	0.1404	−0.1107	0.3729	0.102*	
H25A	0.2417	0.0850	0.4192	0.089*	
H25B	0.2528	0.0072	0.3304	0.089*	
H27	0.1841	0.1615	0.6474	0.090*	
H28	0.0466	0.2738	0.6474	0.113*	
H29	0.0755	0.4063	0.5781	0.116*	
H30	0.2389	0.4253	0.5124	0.094*	
H33	0.3835	0.4212	0.4353	0.083*	
H34	0.5518	0.4173	0.3700	0.100*	
H35	0.6777	0.3059	0.3969	0.097*	
H36	0.6341	0.1984	0.4863	0.074*	
H37	0.5456	0.3708	0.6720	0.068*	
H38	0.5437	0.5029	0.7905	0.086*	
H39	0.4717	0.4734	0.9164	0.106*	
H40	0.4062	0.3111	0.9215	0.090*	
H43	0.4027	0.1483	0.9269	0.072*	
H44	0.3664	−0.0233	0.9164	0.082*	
H45	0.3430	−0.1322	0.7803	0.081*	
H46	0.3536	−0.0662	0.6574	0.075*	

### Atomic displacement parameters (Å^2^)

**Table:**

	*U*^11^	*U*^22^	*U*^33^	*U*^12^	*U*^13^	*U*^23^
Mn1	0.0505 (3)	0.0440 (3)	0.0341 (3)	0.0046 (2)	−0.0015 (2)	0.0154 (2)
Mn2	0.0471 (3)	0.0407 (3)	0.0359 (3)	0.0090 (2)	0.0018 (2)	0.0095 (2)
S1	0.0524 (6)	0.1455 (11)	0.0467 (6)	0.0309 (7)	−0.0036 (5)	0.0284 (6)
O1	0.0569 (15)	0.0507 (14)	0.0521 (14)	−0.0007 (11)	−0.0048 (12)	0.0256 (11)
O2	0.0825 (18)	0.0493 (15)	0.0449 (14)	0.0089 (13)	0.0167 (13)	0.0149 (11)
O3	0.0597 (16)	0.0482 (14)	0.0497 (15)	0.0045 (12)	−0.0011 (12)	0.0039 (11)
O4	0.0553 (15)	0.0653 (16)	0.0523 (15)	0.0215 (13)	−0.0083 (13)	−0.0054 (13)
N1	0.0469 (17)	0.0489 (16)	0.0429 (15)	0.0033 (13)	−0.0004 (13)	0.0213 (13)
N2	0.0480 (17)	0.0450 (16)	0.0534 (17)	−0.0030 (13)	−0.0016 (14)	0.0181 (13)
N3	0.0536 (18)	0.0454 (16)	0.0424 (16)	0.0039 (14)	0.0022 (14)	0.0110 (13)
N4	0.0513 (18)	0.0564 (18)	0.0421 (16)	0.0086 (14)	−0.0009 (14)	0.0143 (14)
N5	0.0507 (19)	0.0496 (18)	0.0605 (19)	0.0115 (14)	0.0005 (15)	0.0104 (15)
N6	0.0583 (19)	0.0474 (17)	0.0388 (15)	0.0097 (14)	0.0016 (14)	0.0132 (13)
N7	0.0552 (18)	0.0423 (16)	0.0392 (15)	0.0055 (14)	0.0002 (13)	0.0056 (12)
N8	0.0599 (19)	0.0438 (17)	0.0477 (17)	0.0105 (14)	0.0107 (14)	0.0133 (13)
C1	0.047 (2)	0.062 (2)	0.059 (2)	−0.0033 (18)	0.0004 (18)	0.0237 (18)
C2	0.042 (2)	0.071 (3)	0.074 (3)	0.0016 (19)	−0.014 (2)	0.021 (2)
C3	0.068 (3)	0.080 (3)	0.058 (2)	0.018 (2)	−0.013 (2)	0.026 (2)
C4	0.065 (3)	0.061 (2)	0.055 (2)	0.009 (2)	−0.005 (2)	0.0289 (19)
C5	0.050 (2)	0.0434 (19)	0.0409 (18)	0.0112 (16)	0.0050 (16)	0.0137 (15)
C6	0.054 (2)	0.0404 (19)	0.0476 (19)	0.0088 (16)	0.0075 (17)	0.0189 (15)
C7	0.072 (3)	0.066 (3)	0.074 (3)	0.001 (2)	0.004 (2)	0.043 (2)
C8	0.081 (3)	0.066 (3)	0.111 (4)	−0.010 (2)	0.012 (3)	0.049 (3)
C9	0.064 (3)	0.060 (3)	0.104 (4)	−0.017 (2)	−0.001 (3)	0.021 (2)
C10	0.061 (3)	0.058 (2)	0.069 (3)	−0.004 (2)	−0.009 (2)	0.020 (2)
C11	0.060 (2)	0.057 (2)	0.055 (2)	0.0036 (19)	0.0045 (19)	0.0151 (18)
C12	0.073 (3)	0.069 (3)	0.067 (3)	0.006 (2)	0.022 (2)	0.008 (2)
C13	0.094 (4)	0.084 (3)	0.051 (3)	−0.002 (3)	0.019 (3)	−0.007 (2)
C14	0.083 (3)	0.080 (3)	0.039 (2)	−0.005 (2)	−0.004 (2)	0.0007 (19)
C15	0.058 (2)	0.0433 (19)	0.0418 (19)	−0.0076 (17)	−0.0034 (17)	0.0088 (15)
C16	0.054 (2)	0.050 (2)	0.045 (2)	−0.0064 (17)	−0.0103 (17)	0.0141 (16)
C17	0.070 (3)	0.099 (3)	0.059 (3)	0.003 (3)	−0.024 (2)	−0.003 (2)
C18	0.058 (3)	0.108 (4)	0.085 (3)	0.002 (3)	−0.029 (3)	0.016 (3)
C19	0.043 (2)	0.079 (3)	0.088 (3)	0.006 (2)	0.002 (2)	0.026 (2)
C20	0.057 (2)	0.072 (3)	0.056 (2)	0.012 (2)	0.004 (2)	0.0209 (19)
C21	0.0364 (18)	0.048 (2)	0.0421 (18)	0.0022 (15)	−0.0001 (15)	0.0174 (16)
C22	0.063 (2)	0.059 (2)	0.045 (2)	0.0128 (19)	−0.0052 (18)	0.0156 (17)
C23	0.065 (3)	0.094 (3)	0.051 (2)	0.028 (2)	0.003 (2)	0.018 (2)
C24	0.059 (3)	0.118 (4)	0.089 (3)	0.002 (3)	−0.010 (2)	0.049 (3)
C25	0.069 (3)	0.068 (3)	0.083 (3)	0.017 (2)	−0.014 (2)	0.012 (2)
C26	0.043 (2)	0.045 (2)	0.058 (2)	0.0043 (16)	−0.0125 (18)	0.0069 (17)
C27	0.055 (3)	0.078 (3)	0.093 (3)	0.013 (2)	0.011 (2)	0.019 (2)
C28	0.052 (3)	0.110 (4)	0.120 (4)	0.026 (3)	0.016 (3)	0.016 (3)
C29	0.075 (4)	0.084 (4)	0.127 (5)	0.042 (3)	−0.011 (3)	0.010 (3)
C30	0.079 (3)	0.067 (3)	0.086 (3)	0.028 (2)	−0.019 (3)	0.012 (2)
C31	0.063 (2)	0.041 (2)	0.049 (2)	0.0140 (17)	−0.0169 (18)	0.0025 (16)
C32	0.069 (2)	0.0375 (18)	0.0372 (18)	0.0042 (17)	−0.0123 (18)	0.0048 (14)
C33	0.097 (3)	0.054 (2)	0.058 (2)	0.002 (2)	−0.022 (2)	0.0227 (19)
C34	0.120 (4)	0.071 (3)	0.064 (3)	−0.018 (3)	0.006 (3)	0.031 (2)
C35	0.096 (4)	0.078 (3)	0.074 (3)	−0.002 (3)	0.027 (3)	0.028 (2)
C36	0.071 (3)	0.062 (2)	0.055 (2)	0.007 (2)	0.013 (2)	0.0138 (19)
C37	0.073 (3)	0.050 (2)	0.045 (2)	−0.0017 (19)	−0.0005 (19)	0.0091 (17)
C38	0.107 (4)	0.047 (2)	0.055 (2)	−0.003 (2)	−0.005 (2)	0.0029 (19)
C39	0.140 (5)	0.059 (3)	0.055 (3)	0.007 (3)	0.001 (3)	−0.009 (2)
C40	0.117 (4)	0.069 (3)	0.039 (2)	0.016 (3)	0.014 (2)	0.0070 (19)
C41	0.055 (2)	0.055 (2)	0.0376 (19)	0.0147 (17)	0.0028 (16)	0.0105 (16)
C42	0.046 (2)	0.055 (2)	0.0430 (19)	0.0115 (16)	0.0055 (16)	0.0165 (16)
C43	0.064 (3)	0.076 (3)	0.048 (2)	0.009 (2)	0.0099 (19)	0.0256 (19)
C44	0.066 (3)	0.087 (3)	0.068 (3)	0.018 (2)	0.015 (2)	0.045 (3)
C45	0.071 (3)	0.057 (2)	0.086 (3)	0.011 (2)	0.022 (2)	0.036 (2)
C46	0.079 (3)	0.050 (2)	0.062 (2)	0.009 (2)	0.015 (2)	0.0160 (19)
Cl1	0.0618 (6)	0.0534 (5)	0.0590 (6)	0.0018 (5)	0.0008 (5)	0.0174 (4)
Cl2	0.0642 (6)	0.0714 (7)	0.0664 (6)	0.0117 (5)	0.0027 (5)	0.0385 (5)
O5	0.0632 (18)	0.083 (2)	0.0770 (19)	0.0122 (15)	−0.0012 (15)	0.0215 (15)
O6	0.118 (3)	0.0532 (17)	0.087 (2)	0.0144 (17)	0.0171 (19)	0.0122 (15)
O7	0.084 (2)	0.085 (2)	0.139 (3)	−0.0133 (18)	0.023 (2)	0.050 (2)
O8	0.114 (3)	0.117 (3)	0.0557 (18)	0.012 (2)	−0.0214 (18)	0.0119 (17)
O9	0.187 (4)	0.077 (2)	0.127 (3)	−0.024 (2)	−0.044 (3)	0.050 (2)
O10	0.122 (3)	0.123 (3)	0.0678 (19)	0.013 (2)	0.0204 (19)	0.0482 (19)
O11	0.132 (3)	0.131 (3)	0.086 (2)	−0.034 (2)	−0.036 (2)	0.062 (2)
O12	0.118 (3)	0.193 (5)	0.207 (5)	0.093 (3)	0.073 (3)	0.117 (4)

### Geometric parameters (Å, °)

**Table:**

Mn1—O2^i^	2.121 (2)	C31—C32	1.475 (5)
Mn1—O1	2.124 (2)	C32—C33	1.391 (5)
Mn1—N1	2.234 (3)	C33—C34	1.378 (6)
Mn1—N4	2.236 (3)	C34—C35	1.349 (6)
Mn1—N3	2.254 (3)	C35—C36	1.369 (5)
Mn1—N2	2.293 (3)	C37—C38	1.362 (5)
Mn2—O4^ii^	2.110 (2)	C38—C39	1.356 (6)
Mn2—O3	2.112 (2)	C39—C40	1.373 (6)
Mn2—N8	2.255 (3)	C40—C41	1.380 (5)
Mn2—N6	2.263 (3)	C41—C42	1.479 (5)
Mn2—N5	2.298 (3)	C42—C43	1.387 (5)
Mn2—N7	2.301 (3)	C43—C44	1.370 (5)
S1—C23	1.807 (4)	C44—C45	1.360 (6)
S1—C24	1.808 (4)	C45—C46	1.378 (5)
O1—C21	1.246 (4)	Cl1—O8	1.420 (3)
O2—C21	1.245 (4)	Cl1—O5	1.420 (3)
O2—Mn1^i^	2.121 (2)	Cl1—O7	1.421 (3)
O3—C26	1.251 (4)	Cl1—O6	1.428 (3)
O4—C26	1.235 (4)	Cl2—O12	1.381 (4)
O4—Mn2^ii^	2.110 (2)	Cl2—O9	1.395 (3)
N1—C1	1.336 (4)	Cl2—O11	1.411 (3)
N1—C5	1.348 (4)	Cl2—O10	1.415 (3)
N2—C10	1.335 (4)	C1—H1	0.9300
N2—C6	1.341 (4)	C2—H2	0.9300
N3—C11	1.332 (4)	C3—H3	0.9300
N3—C15	1.340 (4)	C4—H4	0.9300
N4—C20	1.336 (4)	C7—H7	0.9300
N4—C16	1.342 (4)	C8—H8	0.9300
N5—C27	1.333 (5)	C9—H9	0.9300
N5—C31	1.347 (4)	C10—H10	0.9300
N6—C36	1.328 (5)	C11—H11	0.9300
N6—C32	1.344 (4)	C12—H12	0.9300
N7—C37	1.336 (4)	C13—H13	0.9300
N7—C41	1.344 (4)	C14—H14	0.9300
N8—C46	1.335 (4)	C17—H17	0.9300
N8—C42	1.341 (4)	C18—H18	0.9300
C1—C2	1.370 (5)	C19—H19	0.9300
C2—C3	1.368 (5)	C20—H20	0.9300
C3—C4	1.379 (5)	C22—H22A	0.9700
C4—C5	1.384 (4)	C22—H22B	0.9700
C5—C6	1.475 (5)	C23—H23A	0.9700
C6—C7	1.385 (5)	C23—H23B	0.9700
C7—C8	1.381 (6)	C24—H24A	0.9700
C8—C9	1.359 (6)	C24—H24B	0.9700
C9—C10	1.373 (5)	C25—H25A	0.9700
C11—C12	1.370 (5)	C25—H25B	0.9700
C12—C13	1.365 (6)	C27—H27	0.9300
C13—C14	1.372 (6)	C28—H28	0.9300
C14—C15	1.381 (5)	C29—H29	0.9300
C15—C16	1.479 (5)	C30—H30	0.9300
C16—C17	1.380 (5)	C33—H33	0.9300
C17—C18	1.365 (6)	C34—H34	0.9300
C18—C19	1.358 (6)	C35—H35	0.9300
C19—C20	1.368 (5)	C36—H36	0.9300
C21—C22	1.519 (4)	C37—H37	0.9300
C22—C23	1.475 (5)	C38—H38	0.9300
C24—C25	1.456 (6)	C39—H39	0.9300
C25—C26	1.526 (5)	C40—H40	0.9300
C27—C28	1.390 (6)	C43—H43	0.9300
C28—C29	1.368 (7)	C44—H44	0.9300
C29—C30	1.361 (7)	C45—H45	0.9300
C30—C31	1.382 (5)	C46—H46	0.9300
			
O2^i^—Mn1—O1	97.97 (9)	N7—C37—C38	123.3 (4)
O2^i^—Mn1—N1	87.09 (10)	C39—C38—C37	118.3 (4)
O1—Mn1—N1	93.10 (9)	C38—C39—C40	119.9 (4)
O2^i^—Mn1—N4	95.09 (10)	C39—C40—C41	119.4 (4)
O1—Mn1—N4	98.77 (9)	N7—C41—C40	120.6 (3)
N1—Mn1—N4	167.49 (10)	N7—C41—C42	116.0 (3)
O2^i^—Mn1—N3	166.07 (11)	C40—C41—C42	123.4 (3)
O1—Mn1—N3	90.46 (10)	N8—C42—C43	121.4 (3)
N1—Mn1—N3	103.58 (10)	N8—C42—C41	116.2 (3)
N4—Mn1—N3	72.57 (10)	C43—C42—C41	122.3 (3)
O2^i^—Mn1—N2	84.63 (10)	C44—C43—C42	119.0 (4)
O1—Mn1—N2	165.16 (9)	C45—C44—C43	120.0 (4)
N1—Mn1—N2	72.37 (10)	C44—C45—C46	118.1 (4)
N4—Mn1—N2	95.54 (10)	N8—C46—C45	123.2 (4)
N3—Mn1—N2	90.06 (10)	O8—Cl1—O5	109.5 (2)
O4^ii^—Mn2—O3	98.49 (9)	O8—Cl1—O7	110.9 (2)
O4^ii^—Mn2—N8	91.21 (10)	O5—Cl1—O7	109.49 (19)
O3—Mn2—N8	90.14 (10)	O8—Cl1—O6	108.53 (19)
O4^ii^—Mn2—N6	96.47 (11)	O5—Cl1—O6	109.31 (19)
O3—Mn2—N6	104.81 (10)	O7—Cl1—O6	109.0 (2)
N8—Mn2—N6	161.91 (10)	O12—Cl2—O9	110.6 (3)
O4^ii^—Mn2—N5	165.22 (11)	O12—Cl2—O11	111.5 (3)
O3—Mn2—N5	92.96 (10)	O9—Cl2—O11	107.7 (2)
N8—Mn2—N5	98.12 (11)	O12—Cl2—O10	106.9 (2)
N6—Mn2—N5	71.43 (11)	O9—Cl2—O10	112.1 (2)
O4^ii^—Mn2—N7	90.41 (10)	O11—Cl2—O10	108.0 (2)
O3—Mn2—N7	160.35 (10)	N1—C1—H1	118.2
N8—Mn2—N7	72.09 (10)	C2—C1—H1	118.2
N6—Mn2—N7	91.45 (10)	C3—C2—H2	120.9
N5—Mn2—N7	81.66 (10)	C1—C2—H2	120.9
C23—S1—C24	102.7 (2)	C2—C3—H3	120.2
C21—O1—Mn1	135.9 (2)	C4—C3—H3	120.2
C21—O2—Mn1^i^	131.8 (2)	C3—C4—H4	120.3
C26—O3—Mn2	137.2 (2)	C5—C4—H4	120.3
C26—O4—Mn2^ii^	130.1 (2)	C8—C7—H7	120.3
C1—N1—C5	118.4 (3)	C6—C7—H7	120.3
C1—N1—Mn1	122.8 (2)	C9—C8—H8	120.2
C5—N1—Mn1	115.7 (2)	C7—C8—H8	120.2
C10—N2—C6	119.2 (3)	C8—C9—H9	120.8
C10—N2—Mn1	125.3 (2)	C10—C9—H9	120.8
C6—N2—Mn1	114.9 (2)	N2—C10—H10	118.6
C11—N3—C15	118.8 (3)	C9—C10—H10	118.6
C11—N3—Mn1	123.2 (2)	N3—C11—H11	118.6
C15—N3—Mn1	117.3 (2)	C12—C11—H11	118.6
C20—N4—C16	118.6 (3)	C13—C12—H12	120.6
C20—N4—Mn1	123.6 (2)	C11—C12—H12	120.6
C16—N4—Mn1	117.7 (2)	C12—C13—H13	120.5
C27—N5—C31	118.7 (3)	C14—C13—H13	120.5
C27—N5—Mn2	123.7 (3)	C13—C14—H14	120.2
C31—N5—Mn2	116.7 (2)	C15—C14—H14	120.2
C36—N6—C32	119.0 (3)	C18—C17—H17	119.9
C36—N6—Mn2	122.4 (2)	C16—C17—H17	119.9
C32—N6—Mn2	118.6 (2)	C19—C18—H18	120.2
C37—N7—C41	118.6 (3)	C17—C18—H18	120.2
C37—N7—Mn2	124.6 (2)	C18—C19—H19	121.0
C41—N7—Mn2	115.8 (2)	C20—C19—H19	121.0
C46—N8—C42	118.2 (3)	N4—C20—H20	118.3
C46—N8—Mn2	122.5 (2)	C19—C20—H20	118.3
C42—N8—Mn2	117.8 (2)	C23—C22—H22A	108.6
N1—C1—C2	123.5 (3)	C21—C22—H22A	108.6
C3—C2—C1	118.2 (4)	C23—C22—H22B	108.6
C2—C3—C4	119.6 (3)	C21—C22—H22B	108.6
C3—C4—C5	119.4 (3)	H22A—C22—H22B	107.6
N1—C5—C4	121.0 (3)	C22—C23—H23A	109.5
N1—C5—C6	116.4 (3)	S1—C23—H23A	109.5
C4—C5—C6	122.6 (3)	C22—C23—H23B	109.5
N2—C6—C7	120.6 (3)	S1—C23—H23B	109.5
N2—C6—C5	116.3 (3)	H23A—C23—H23B	108.1
C7—C6—C5	123.1 (3)	C25—C24—H24A	108.3
C8—C7—C6	119.3 (4)	S1—C24—H24A	108.3
C9—C8—C7	119.6 (4)	C25—C24—H24B	108.3
C8—C9—C10	118.5 (4)	S1—C24—H24B	108.3
N2—C10—C9	122.7 (4)	H24A—C24—H24B	107.4
N3—C11—C12	122.7 (4)	C24—C25—H25A	109.4
C13—C12—C11	118.8 (4)	C26—C25—H25A	109.4
C12—C13—C14	119.1 (4)	C24—C25—H25B	109.4
C13—C14—C15	119.6 (4)	C26—C25—H25B	109.4
N3—C15—C14	121.0 (4)	H25A—C25—H25B	108.0
N3—C15—C16	115.7 (3)	N5—C27—H27	118.8
C14—C15—C16	123.4 (3)	C28—C27—H27	118.8
N4—C16—C17	120.1 (4)	C29—C28—H28	121.0
N4—C16—C15	116.1 (3)	C27—C28—H28	121.0
C17—C16—C15	123.7 (3)	C30—C29—H29	119.9
C18—C17—C16	120.2 (4)	C28—C29—H29	119.9
C19—C18—C17	119.7 (4)	C29—C30—H30	120.3
C18—C19—C20	118.0 (4)	C31—C30—H30	120.3
N4—C20—C19	123.4 (4)	C34—C33—H33	120.5
O2—C21—O1	124.7 (3)	C32—C33—H33	120.5
O2—C21—C22	116.0 (3)	C35—C34—H34	120.2
O1—C21—C22	119.2 (3)	C33—C34—H34	120.2
C23—C22—C21	114.5 (3)	C34—C35—H35	120.5
C22—C23—S1	110.6 (3)	C36—C35—H35	120.5
C25—C24—S1	115.8 (3)	N6—C36—H36	118.6
C24—C25—C26	111.4 (4)	C35—C36—H36	118.6
O4—C26—O3	124.4 (3)	N7—C37—H37	118.4
O4—C26—C25	116.0 (3)	C38—C37—H37	118.4
O3—C26—C25	119.6 (3)	C39—C38—H38	120.9
N5—C27—C28	122.4 (4)	C37—C38—H38	120.9
C29—C28—C27	118.1 (5)	C38—C39—H39	120.1
C30—C29—C28	120.1 (4)	C40—C39—H39	120.1
C29—C30—C31	119.4 (5)	C39—C40—H40	120.3
N5—C31—C30	121.2 (4)	C41—C40—H40	120.3
N5—C31—C32	116.2 (3)	C44—C43—H43	120.5
C30—C31—C32	122.5 (4)	C42—C43—H43	120.5
N6—C32—C33	120.5 (4)	C45—C44—H44	120.0
N6—C32—C31	116.0 (3)	C43—C44—H44	120.0
C33—C32—C31	123.5 (4)	C44—C45—H45	120.9
C34—C33—C32	119.0 (4)	C46—C45—H45	121.0
C35—C34—C33	119.7 (4)	N8—C46—H46	118.4
C34—C35—C36	119.0 (5)	C45—C46—H46	118.4
N6—C36—C35	122.8 (4)		

Symmetry codes: (i) −*x*, −*y*, −*z*; (ii) −*x*+1, −*y*, −*z*+1.
